# NanoBioAnalytical (NBA) Platform to Decipher Extracellular Vesicles Secreted by Microvascular Endothelial Cells Under Benzo[a]pyrene Exposure

**DOI:** 10.3390/bios15020103

**Published:** 2025-02-11

**Authors:** Geetika Raizada, Joan Guillouzouic, Alain Rouleau, Eric Lesniewska, Eric Le Ferrec, Céline Elie-Caille, Wilfrid Boireau

**Affiliations:** 1 Université Marie et Louis Pasteur, CNRS, Institut FEMTO-ST, 25030 Besançon, France; 2 Univ Rennes, Inserm, EHESP, Irset (Institut de Recherche en Santé Environnement et Travail), UMR_S 1085, 35000 Rennes, France; 3 ICB UMR 6303 CNRS, University Bourgogne Europe, 21078 Dijon, France

**Keywords:** extracellular vesicles (EVs), cytotoxicity, polycyclic aromatic hydrocarbons (PAH), surface plasmon resonance imaging (SPRi), atomic force microscopy (AFM)

## Abstract

Recent advances in the clinical extracellular vesicles (EVs) field highlight their potential as biomarkers for diverse diseases and therapeutic applications. This study provides an in-depth characterization of 10k EVs from human microvascular endothelial cells (HMEC-1) exposed to benzo[a]pyrene (B[a]P), a polycyclic aromatic hydrocarbon found in food and smoke. Given EVs’ complexity, with numerous surface and cargo proteins, phenotyping remains challenging. Here, we introduce a multiplex biosensor, in µarray format, for profiling EVs from distinct cellular conditions, employing a multimodal approach that combines surface plasmon resonance imaging (SPRi) and in situ atomic force microscopy (AFM) to decipher EVs’ biochemical and biophysical properties. SPRi experiments showed notable EV capture differences on ligands such as Anti-CD36, Anti-CD81, and Anti-ApoA between treated and control conditions, likely due to B[a]P exposure. A complementary AFM study and statistical analyses revealed size differences between EVs from treated and control samples, with ligands like Annexin-V, Anti-CD36, and Anti-VEGFR1 emerging as ligands specific to potential cytotoxicity biomarkers. Our findings suggest that B[a]P exposure may increase EV size and alter marker expression, indicating phenotypic shifts in EVs under cytotoxic stress. The original combination of SPRi and AFM reveals valuable data on the phenotypical and morphological heterogeneities of EV subsets linked to cytotoxic stresses and highlights the potential of EVs as specific toxicological markers.

## 1. Introduction

Extracellular vesicles (EVs) are proteo-lipidic membranous particles released into extracellular environments from almost all types of cells. They are known to play an important role in intercellular communication and transfer the material from the originating cells to the host cells [1,2]. Because of their presence in various biological fluids including but not limited to blood, urine, saliva, and cerebrospinal fluid, they are currently explored for their potential application as biomarkers in various pathologies. They are known to contain nucleic acids, lipids, proteins, and in some cases organelles in their cargo [3,4]. This versatility has sparked significant interest in their use as biomarkers for toxicant exposure, as EVs can reflect cellular responses to environmental contaminants and provide insights into the mechanisms of toxicity. This potential has driven research into the role of EVs in responding to environmental pollutants, particularly those with widespread exposure and significant health impacts [5]. Among these, B[a]P, a polycyclic aromatic hydrocarbon (PAH), has emerged as a key focus due to its well-documented cytotoxic, mutagenic, and carcinogenic properties [6]. Exposure to B[a]P induces cytotoxicity through the formation of protein and DNA adducts, as well as oxidative stress resulting from the overproduction of reactive oxygen species (ROS) [7]. The toxic cellular environment induced by B[a]P triggers complex responses that extend beyond the directly affected cells, impacting neighboring cells through alterations in the cellular microenvironment [5]. EVs released from affected cells may carry oxidized lipids, altered proteins, and damaged or modified DNA, potentially exerting harmful effects on recipient cells [8]. EVs may also serve a protective role for the originating cells; however, they can simultaneously propagate damage to neighboring cells through a bystander effect by transferring modified proteins, DNA adducts, or bioactive metabolites [9]. EVs are central to the cellular response to oxidative stress, playing a critical role in balancing cellular homeostasis and propagating damage within the microenvironment. This dynamic response gives rise to diverse biogenesis mechanisms, altered protein expression, and ultimately, the formation of distinct EV subpopulations.

Several technologies like Western Blot, ELISA, and Flow Cytometry are commonly used for EV detection and characterization [10]. While these techniques are reliable, they often come with challenges such as being time-consuming, requiring large sample volumes, and involving intricate labeling procedures. In order to overcome these limitations and to address the discrimination of small changes in the different EV subpopulations, there is a growing interest in and trend for label-free detection methods in a multiplex format. Combining various biophysical techniques has proven effective in analyzing and identifying EV subpopulations that are often overshadowed [11]. Techniques such as optical sensors, including plasmonic and interferometric sensing, as well as electrochemical sensors like potentiometry, potentiostatic, glavanostatic, and impedance spectroscopy, are gaining prominence for their ability to enable the efficient and rapid analysis of EVs. The main principle behind these methods is based on biomolecular interactions between biologic or synthetic receptors—such as antibodies, aptamers or chemical ligands—and EV surfaceome for the specific capture of EV subpopulations [12].

Surface plasmon resonance (SPR), a well-established optical label-free biosensing technology, enables the study of molecular interactions between immobilized biomolecules and biological targets with high sensitivity at the vicinity of the gold surface (<250 nm), making it an ideal tool for EV characterization [13]. The multiplexing provided by this technique also allows simultaneous EV subpopulation detection and quantification by using a panel of surface-immobilized antibodies [14]. Several studies have demonstrated the usefulness and relevancy of SPRi biosensors for detecting various diseases, including cancer and Alzheimer’s disease, through EV analysis [15,16,17]. Due to the complex composition and heterogeneous nature of EVs, it is of interest to combine SPR with other technologies to improve their qualification. Hsu et al. integrated SPR with surface plasmon-enhanced fluorescence spectroscopy (SPEFS) to simultaneously detect protein and miRNA content in tumor-derived EVs [18]. In our group, Obeid et al. have previously developed a nano-bio-analytical (NBA) platform which combined SPRi with AFM and mass spectrometry, where they analyzed thrombin-activated platelet EVs related to transfusion safety [19]. However, a significant challenge in detecting EV subpopulation lies in the expression of hundreds of proteins on their surfaces, adding complexity to their characterization.

In this study, we selected different proteins known to be present on the surface of EVs produced by endothelial cells, with the aim of determining whether our approach allows us to detect them and whether the EVs produced by endothelial cells in the presence of B(a)P exhibit a modified ‘surfaceome’. Building on the NBA platform previously developed in our group [19,20], we employed a multimodal approach combining SPRi and AFM. SPRi facilitated the identification of EV phenotypes by using specific ligands (antibodies and proteins) grafted onto a biochip, enabling immunocapture while maintaining EV structural integrity. To address the complexity posed by the vast array of surface proteins, we expanded the range of ligands for selective EV capture by miniaturizing the ligand spots on the biochip. This was accomplished through the implementation of automatic nano-spotting, greatly enhancing the multiplexing capacity. Complementing this, AFM provided precise metrological data on the selectively captured EVs, linking surface protein expression to their morphological and structural features, thereby enabling a highly discriminative characterization of EV subpopulations.

## 2. Materials and Methods

### 2.1. Cell Culture and EV Isolation

The method of cell culture and EV isolation was described by Le Goff et al. [21]. Human microvascular endothelial cell line (HMEC-1) was sourced from the Center for Disease Control and Prevention, Atlanta, GA, USA; the cells were cultured in endothelial basal medium MCDB131 (US Biological Life Sciences, Ref E3000-01G, Salem, MA, USA) which contained 10% heat deactivated (56 °C, 30 min) fetal bovine serum (FBS, Dutscher, Ref 500105A1A, Bernolsheim, France), L-glutamine (Gibco, Ref 25030, 10 mM final, Paisley, Scotland, UK), penicillin/streptomycin (Gibco, Ref 15140-122, 100 unit/mL final), gentamycin (Gibco, Ref 15750-037, 500 µg/mL final), epithelial growth factor (EGF, Sigma, Ref E9644, 10 ng/mL final, Molsheim, France), and hydrocortisone (Up John, Ref 3400932141159, 1 µg/mL final, Paris, France) in 151.9 cm^2^ petri dishes (Corning, Ref 353025, Borre, France). The medium’s pH was adjusted to 7.6 using sodium bicarbonate (Gibco, Ref 25080-060) and replaced every 2–3 days. Cells were passaged weekly through trypsinization (trypsin EDTA 0.05%, Gibco, Ref 25300-054). Upon reaching 90% confluence, the cells were incubated overnight in FBS-free medium. The following day, they were treated with 100 nM B[a]P (Sigma, Ref B1760) dissolved in DMSO (Sigma, Ref D8418), ensuring the final DMSO concentration did not exceed 0.0005% (*v*/*v*) (treated condition). Control cultures received the same volume of DMSO without B[a]P (control condition).

For EV isolation, international guidelines for EV isolation were followed [22]. Conditioned media were centrifuged at 3650× *g* for 10 min at 4 °C to remove cells and debris. The clarified supernatant was transferred into ultracentrifugation tubes (Beckman Coulter, 5/8 × 4 P.A tube, 17 mL, Ref.: 337986, Indianapolis, IN, USA). To adjust the volume as needed, sterile 1X PBS verified as EV-free by Nanoparticle Tracking Analysis (Gibco, DPBS 1X, Ref.: 14190-094) was added. EVs were pelleted by ultracentrifugation at 10,000× *g* for 30 min at 4 °C using an Optima L-90 K ultracentrifuge with an Sw 28.1 Ti rotor or an XE-90 ultracentrifuge with an Sw 32 Ti rotor (Beckman Coulter, Indianapolis, IN, USA).

After removing the supernatants, the 10k EV pellets were washed by resuspension and, if required, pooled into a single ultracentrifugation tube per treatment condition. Following volume adjustment with 1X PBS, a second ultracentrifugation step was performed at 10,000× g for 30 min at 4 °C. The final 10k EV pellet was resuspended in 50 μL of 1X PBS and stored at −20 °C. EV concentration was determined using Nanoparticle Tracking Analysis (NanoSight NS300, Malvern Instruments, Worcestershire, UK) following a protocol of five 60 s video recordings. Since the 10k EV pellet contains both small and large EVs (lEVs), we have used the term “10k EVs” from this point onwards to reflect this inclusivity.

### 2.2. SPRi Biochip Preparation

SPRi biochips were fabricated by FEMTO Engineering in the MIMENTO clean room facility (Besançon, France) following the previously described method [20,23]. These biochips were composed of glass slides (SF11) with a thin coating of gold (48 nm) and titanium (2 nm) as an adhesive layer in between. The biochips were then chemically functionalized by incubating the mixture of mercapto-1-undecanol (11-MUOH: C11) and mercapto 1-hexadecanoic acid (16-MHA: C16) overnight with the ratio of 80:20 by mole. This was followed by the activation of the biochip using EDC-NHS chemistry by incubating the biochip in a mixture of 200 mM ethyl (dimethylaminopropyl) carbodiimide/N hydroxysuccinimide (EDC) and 50 mmol/L N-hydroxysuccinimide (Sulfo-NHS) for 30 min in the dark at room temperature. This step facilitated the activation of NHS esters, enabling the covalent grafting of ligand proteins, including antibodies and the passivation agent (Rat Serum Albumin, RSA), by forming a bond between the amine (-NH_2_) groups and the NHS esters.

### 2.3. Automated Ligand Spotting and Biochip Pattern

After activating the biochip, the next step was to graft the chosen ligands (Table 1). We employed the nano-spotter system (sciFLEXARRAYER by *Scienion,* Berlin, Germany) to deposit small volumes of ligands at the designated place. This system operates on the principle of non-contact liquid dispensing via a piezo dispense capillary (PDC). For the experiments, PDC70 was utilized with the following parameters: 91 V, 47 µs pulse duration, 500 Hz frequency, and 200 µs LED delay. A volume of 3 nl was deposited for each spot.

**Figure 1:**
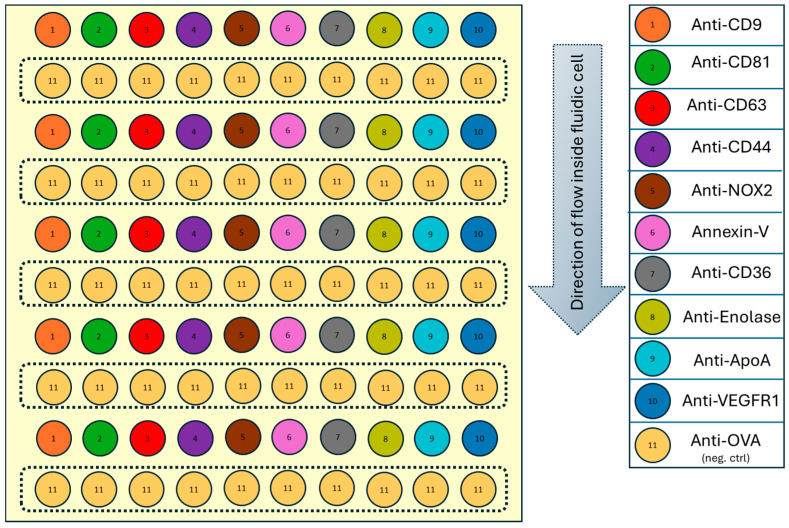
Biochip design with 100 miniaturized spots of 11 different ligands.

The following pattern (Figure 1) of 100 spots (10 rows × 10 columns) was printed, with each spot measuring approximately 200 µm in diameter and spaced 600 µm apart.

The negative control is ensured by an irrelevant IgG directed against ovalbumin as previously published [20]. Anti-OVA was alternated as a negative control, while ligands of interest were arranged in vertical rows to minimize spotting time and account for mass transfer effects, ensuring consistent sample availability across the biochip.

### 2.4. Immunocapture Experiments: SPRi

The subsequent steps following ligand grafting are thoroughly detailed in our previous work [20] and are all monitored inside the SPR apparatus ensuring very high quality controls. The immunocapture SPRi experiment can be divided into two main phases: (i) passivation with RSA and deactivation of the biochip with Ethanolamine, and (ii) kinetic monitoring of sample interactions. The uniformity of antibody grafting was assessed by the level of RSA grafting during the passivation step; this step acts as a quality check for all the biochips used in this study (Supplementary Information Figures S1 and S2).

All the experiments were conducted based on the Horiba SPRi Plex II system, using running buffer HEPES 10 mM (Sigma-Aldrich, H3375-100G) + CaCl_2_ 5 mM (Sigma-Aldrich, 223506-500G) buffer solution. Samples containing EVs (concentration ~ 10^8^ particles/mL) were injected at 10 µL/min for 20 min. After observing the sample interaction with different ligands and having a stable baseline, glutaraldehyde (0.5%) was injected at 20 µL/min for 10 min to fix the EVs captured on the biochip. This fixed biochip was then further characterized with AFM for obtaining the size profile and density of EVs captured.

### 2.5. AFM Characterization

AFM imaging was conducted using the Bruker Nanowizard^®^4 Bioscience system (Berlin, Germany) in tapping mode (AC mode) in air, employing triangular Pyrex-Nitride AFM tips (spring constant: 0.08 N/m). After aligning the laser on the AFM tip and optimizing the SUM value, we calibrated the tip in air before engaging the sample surface. Using the AC Feedback mode wizard, we performed a frequency sweep (Lock-in Phase vs. Frequency) to determine the optimal drive frequency and set point. By specifying the drive amplitude (typically around 20 nm) and the frequency range, we identified the best settings for stable oscillation and precise feedback control, usually setting the set point to 70–80% of the drive amplitude. This ensured reliable operation for high-quality imaging or measurements. During the scanning, further adjustments were made as necessary. Images were captured at a line rate of 1 Hz with a resolution of 512 × 512 pixels. Initial scans covered a 10 × 10 µm area to examine the surface, followed by detailed 2 × 2 µm scans used for particle counting and size profiling.

Image analysis was carried out using Mountain’s SPIP 9 software, utilizing the “Particle Analysis” function to measure object counts and profile their size based on maximum diameter and height. To ensure that only EVs adhered to the surface were analyzed, a dynamic height threshold of 8.5 nm was applied, excluding objects smaller in height than 8.5 nm, and an additional projected area threshold was implemented to disregard objects with areas smaller than 200 nm^2^. These filtering steps effectively isolated EVs in the AFM images, ensuring precise size and count measurements [24].

### 2.6. Statistical Analysis

Python 3.9 code was written to extract the data and obtain different curves like plasmon curves, kinetic data, and response obtained at different families of ligands. Afterwards, the plasmon curves were analysed to quantify ligand grafting by measuring the angular shift between the ligand and the biochip surface. An alternative method involved assessing the reactivity of RSA during the passivation step, where ambiguous results were excluded by verifying that a significant amount of RSA was not grafted onto the ligand, indicating proper ligand grafting. To minimize the impact of non-specific interactions, the SPRi response for each ligand was adjusted by subtracting the average response from negative control ROIs.

To ensure consistency across experiments, SPRi responses for different ligands were normalized to account for sample variability and preparation inconsistencies. The reference for normalization was selected based on its low variability, determined as the ratio of the standard deviation to the mean response across experiments. Anti-CD44, which exhibited the most stable and reproducible response with ratios of 0.48 and 0.43 for control and treated conditions, respectively, was chosen as the normalization reference. This was based on its low variability, calculated as the ratio of the standard deviation to the mean response across experiments. Responses for other ligands were normalized by dividing their values by the response of Anti-CD44, which consistently maintained a fixed value in the phenotype profile. This normalization approach provided a reliable baseline for comparing ligand interactions, reducing variability, and enhancing the accuracy of the data.

To explore differences between the two conditions using AFM data, we conducted a *Mann-Whitney U* test to assess whether the means of the two groups were statistically different. This test compares two independent datasets which are not normally distributed and whose variances are unequal. It ranks all data points and verifies if the ranks in one dataset are significantly different from another dataset. It evaluates the null hypothesis (H_0_), which assumes no significant difference between the groups, against the alternative hypothesis (H_1_), which posits a significant difference. A *p*-value less than 0.05 indicates rejection of the null hypothesis, confirming a significant difference. The *Mann-Whitney U* test was performed for each ligand to compare AFM data between the control and treated conditions, identifying whether significant differences in EV capture were present for specific ligands. The analysis was implemented using the “*scipy.stats*” module and the “*mannwhitneyu*” function in Python 3.9, ensuring robust statistical evaluation of the data.

## 3. Results

### 3.1. Kinetic Monitoring of Sample Interactions

The immunocapture experiments were conducted on the sample originating from both control and treated conditions. The study aimed to evaluate the differences in ligand interactions with samples from two conditions, enabling a comparative analysis under consistent experimental parameters. CD81, CD63, and CD9 targets were selected as markers for ubiquitous EVs. Annexin-V was chosen for its ability to bind phosphatidylserine (PS), which is present on microvesicles. CD44, CD36, and VEGFR1 targets were used as markers for endothelial cells. NOX2 and Enolase targets, while expressed in endothelial cells, were included due to their involvement in toxic mechanisms associated with exposure to environmental contaminants. Anti-ApoA was included in our chip pattern to detect Apoliprotein A, and Anti-OVA as a negative control.

**Figure 2:**
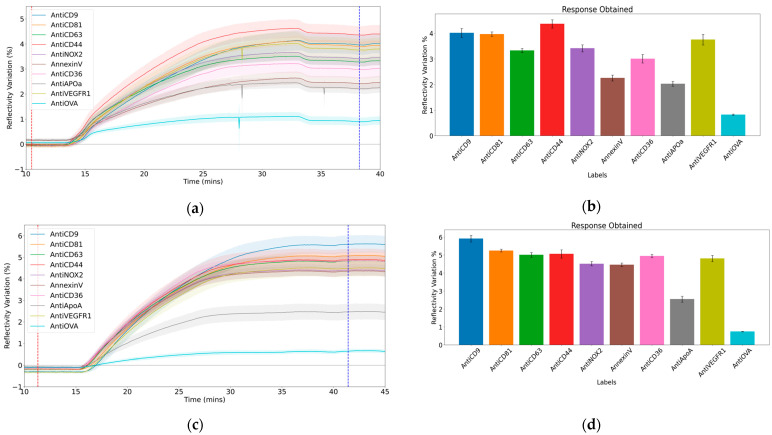
Sensorgrams and responses measured on different ligands following 10k EV sample injection (10^8^ EVs/mL at 10 µL/mL). (**a**) Sensorgram from one of the experiments conducted under the control condition and (**b**) the corresponding response. (**c**) Sensorgram from a sample injection (from one experiment) under the treated condition and (**d**) the calculated response. The shaded areas in the sensorgrams represent the standard deviation across different spots for a given ligand. The red and blue dotted lines indicate the start and end points used to calculate the response for each ligand.

Figure 2a,c illustrate the kinetic curves from two experiments conducted on samples from the control and treated conditions, respectively, depicting the variation in reflectivity (%) over time (minutes) during sample injection (10^8^ particles/mL). An initial increase in reflectivity is observed, followed by distinct interaction patterns as the injection continues, with each ligand family exhibiting unique interactions with the EVs. (Note: Anti-Enolase was excluded from the analysis due to its lack of response which raised uncertainty about its specificity and rendered it unsuitable for the study). The response (Figure 2b,d) was calculated using the formula “End signal—Start signal,” where the end signal represents the stabilized reflectivity variation after the injection, and the start signal denotes the reflectivity variation before the injection began. These points are marked by the dotted blue and red lines in Figure 2a and Figure 2c, respectively.

Each ligand family was analysed using five spots, while the negative control included a total of 50 spots, providing robust statistical data for comparison. The low standard deviation observed for the negative control confirmed the reliability of the experimental setup and the measured responses.

Multiple experiments were conducted for each condition, and those with consistent parameters, such as identical sample concentrations, were selected for analysis. This resulted in the inclusion of five experiments for the control condition and four for the treated condition. The responses from the ligands were highly specific, showing a difference of over 200% compared to the negative control, demonstrating effective ligand-based capture.

#### On-Chip Phenotype Profiling

**Figure 3:**
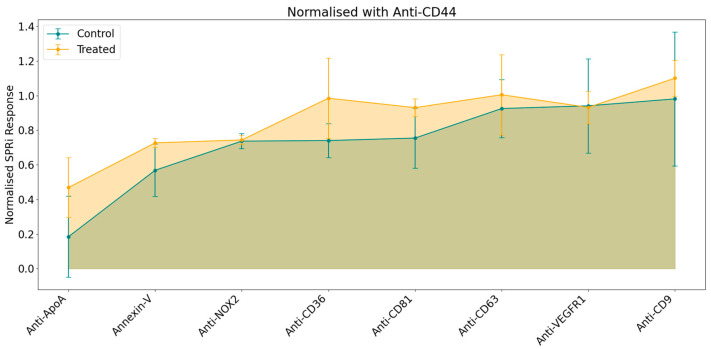
Normalized SPRi responses obtained on a multiplexed biochip for large EV samples originating from control (blue) and treated (orange) conditions.

To generate the phenotype profile, the responses were calculated as previously described (see Section 2.6), followed by normalization using a statistical approach to ensure comparability across experiments. The resulting profile was derived from the normalized average SPRi response for each ligand, as illustrated in Figure 3.

The general pattern in the phenotype profile indicated a stronger SPRi response in samples from the treated condition despite injecting similar concentration of EVs (10^8^ particles/mL) for both samples. Among the panel of selected ligands, the most notable variations were observed with Anti-CD36, Anti-CD81, and Anti-ApoA; a lower degree was observed for Annexin-V, Anti-CD63, and Anti-CD9. Conversely, no differences were detected in the responses for Anti-NOX2 and Anti-VEGFR1. The multiplexed SPRi analysis on 10k EV samples shows a stronger phenotype profile related to B[a]P on CD36, CD81, and ApoA targets.

### 3.2. AFM Data: Metrology

Following SPRi analysis, according to the hyphenated procedure originated in the team, the conditioned biochips were characterized with the help of AFM. This technique offers high-resolution imaging, enabling detailed visualization of variations in EV morphology and size across different conditions. The resulting images can reveal differences in particle number, size, shape, or surface characteristics, which may correlate with the SPRi response variations or provide novel insights. These insights could include changes in EV topology or dimensions, such as height or maximum diameter, which could potentially be attributed to the presence of B(a)P in the culture medium during their production by the cells.

**Figure 4:**
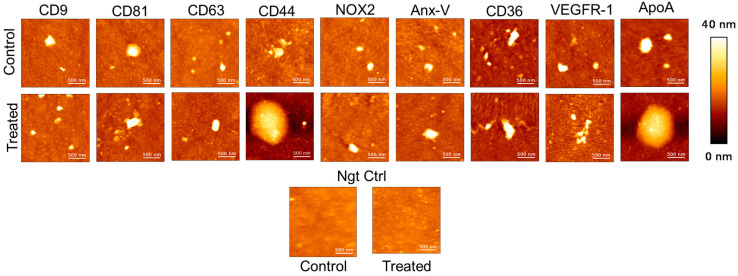
AFM topological images obtained on the EVs captured at different ligands originating from control and treated conditions. The two last images at the bottom correspond to the images registered on a spot composed of the irrelevant anti-OVA IgG as negative control (with the z-scale 40 nm for all the images).

The following AFM topographical (height) images (Figure 4) were obtained at different ligands spotted on the biochip, revealing that EVs generally exhibit a round or elliptical shape. Elliptical morphology may result from interactions between the antibodies and the EVs, potentially leading to their elongation and spreading. Notably, a small proportion of large EVs (above 200 nm in diameter) were observed alongside the predominance of small EVs across all ligands. During the scanning of negative control spots, some objects were detected, likely arising from non-specific interactions. However, their density was significantly lower compared to the ligand-specific images. AFM images of the ligand spots were captured at a size of 2 × 2 µm^2^, while those of the negative control were taken at 10 × 10 µm^2^. This disparity in object density aligns with and further supports the SPRi response observed. The number of AFM images captured per ligand varied, depending on the number needed to ensure the acquisition of at least 100 EVs per ligand.

The size profile of the whole pool of EVs per ligand was obtained using the “*Particle Analysis*” module in *Mountain’s SPIP 9* software. Parameters such as maximum diameter and height were analysed to examine differences between EVs derived from the two conditions (Supplementary Information Figure S3). This global analysis highlights the great diversity of extracellular vesicles (originated from control or treated cells conditions) selectively enriched on-chip by bio-recognition processes and nano-characterized at the single EV level by AFM.

**Figure 5:**
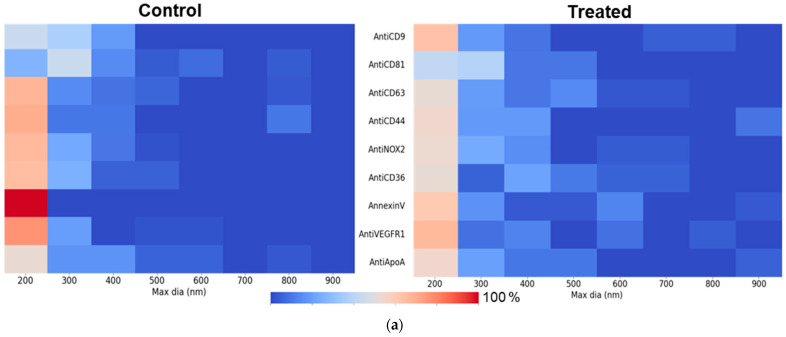

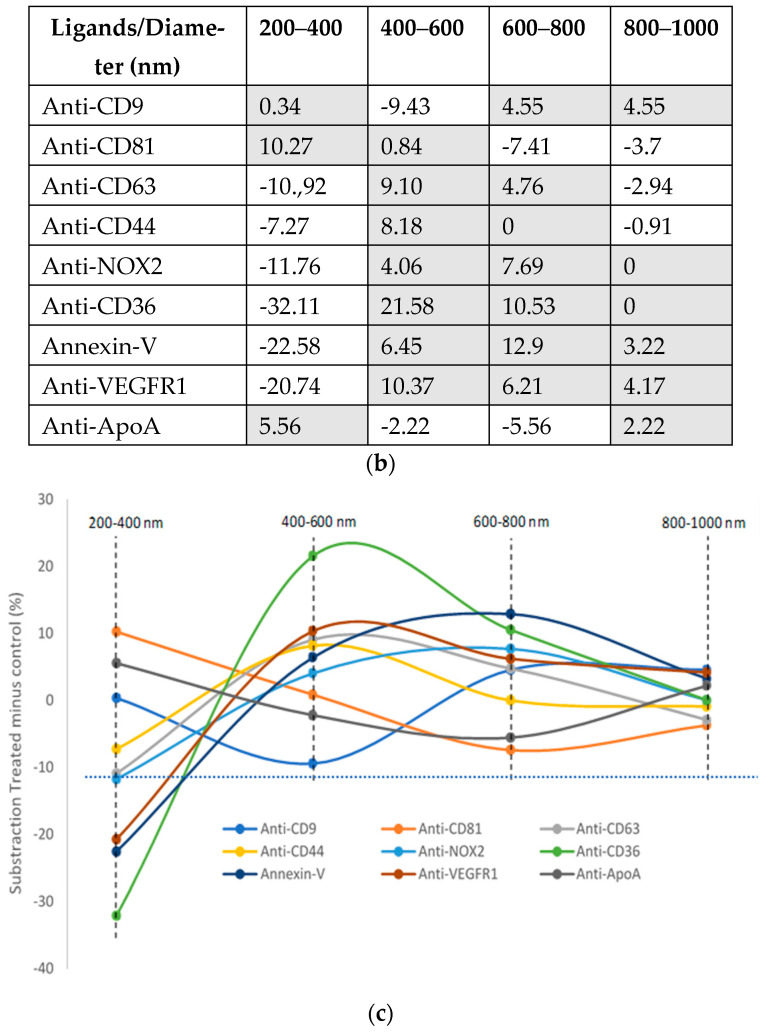
Size distribution of lEVs with diameter > 200 nm, captured on the different ligands: (**a**) Heat maps illustrating the size distribution of EVs (maximum diameter > 200 nm) captured on various ligands. The bin size for these heatmaps is set at 100 nm, with each bin representing the EVs falling within the size range specified on the x-axis. The color scale represents the percentage of particles, ranging from 0 to 100%. (**b**) Quantification of the evolution of lEVs’ maximum diameter with the B(a)P treatment. (**c**) Graphical representation of maximum diameter values, as presented in the table, between treated and control conditions.

Following the global analysis of EVs captured on various ligands, we decided to refine the investigation on large EVs naturally present in the 10k EV pellet. The resulting heat maps (Figure 5a) reveal, for several ligands, a broader distribution of EV sizes in the treated condition, indicating a shift toward large EVs. The shift is further supported by the difference observed between the treated and control conditions in various size ranges, as shown in Figure 5b, which takes into account the relative size evolution of EVs, for each ligand. Indeed, the percentage of large EVs has been determined in the windows “200–400”, “400–600”, “600–800” and “800–1000” nm, in maximum diameter, in the two conditions (control and treated). A negative value means that there were more lEVs in the control condition. A positive value (highlighted in grey in the table) means that there are more lEVs in the treated condition.

The graph (Figure 5c) shows tendencies of lEV biogenesis under pollutant exposure in the function of the ligand of capture. Based on this representation by maximum diameter size, we clearly discriminate several subpopulations of EVs that reveal a broader distribution of EV sizes in the treated condition, especially those presenting an on-chip phenotype CD36^+^, PS^+^, VEGFR1^+^, CD63^+^, NOX2^+^, and CD44^+^ and, to a lesser extent, CD9^+^, which is only affected on the largest sizes.

We applied a Mann-Whitney U analysis to lEVs with a maximum diameter greater than 200 nm to statistically evaluate the relevance of the set of subpopulations revealed by the heat map while including a second parameter (height). From this analysis, we expected significant differences in size between the two conditions, control and treated, and the confirmation of the broader effect induced by B[a]P exposure. The results, summarized in Table 2, revealed notable differences for specific markers.

Anti-CD36 displayed significant differences in both “Maximum Diameter” and “Height”, whereas Anti-VEGFR1, Annexin-V, Anti-CD9, and Anti-CD81 showed significant differences in one parameter either in “Height” or in “Maximum Diameter”. In addition, some ligands, including Anti-CD44, Anti-NOX2, Anti-ApoA, and Anti-CD63, did not exhibit any significant differences between the two conditions. This analysis underscores the targeted impact of treatment response across different EV subpopulations and ligands. By confronting both investigations, it appears clear that each lEV subpopulation bearing an on-chip phenotype including CD36^+^, PS^+^, VEGFR1^+^, and CD9^+^ are the most promising potential biomarkers of B[a]P toxicity.

## 4. Discussion

Through this biophysical study, we obtained insights into EV subpopulation properties under different conditions, revealing distinct patterns linked to specific surface protein expression. By employing the NBA platform which comprises advanced analytical techniques such as AFM and SPRi, coupled with statistical evaluations, we have characterized EV subpopulations, focusing on ligand-specific interactions and size-related parameters. This comprehensive approach has allowed us to uncover treatment-induced changes in EV ligand binding and EV size, shedding light on the underlying biological mechanisms and the potential diagnostic value of EVs in distinguishing between conditions.

Beyond the general trend of a higher SPRi response for treated EV samples, significant differences in EV capture were observed for the ligands Anti-CD36, Anti-CD81, and Anti-ApoA, which could be correlated to cellular response to B[a]P exposure. CD36 plays a vital role in scavenging damaged or dying cells, regulating inflammation, and modulating pro-apoptotic and anti-angiogenic signalling pathways. It is crucial in managing cellular stress responses by recognizing apoptotic markers, such as phosphatidylserine, on the surface of damaged cells [25,26]. The elevated response observed with Anti-CD36 suggests the presence of CD36+ EVs, potentially reflecting cellular mechanisms to clear apoptotic debris and regulate inflammatory responses triggered by cytotoxic stress induced by pollutant exposure. AFM observations further support this, showing a broader distribution of large EVs in the treated condition, particularly for Anti-CD36, indicating treatment-induced changes in EV morphology.

The increased response observed for Anti-CD81 and to a lesser extent for Anti-CD63 and Anti-CD9 in the treated condition compared to controls may be attributed to the elevated production of vesicles under cytotoxic stress. Alternatively, it is also possible that these markers are overexpressed on EVs from the treated condition, leading to higher interaction values.

Interestingly, the broader size distribution towards larger EVs captured on ligands like Anti-CD36, Annexin-V, and Anti-VEGFR1 further corroborates the generation of certain larger EVs in the treated condition, reflecting treatment-induced effects on EV size and abundance. Moreover, classical ubiquitous EV markers, such as Anti-CD9, Anti-CD63, and Anti-CD81, displayed relative consistent size distributions across conditions, highlighting the stability of these EV subpopulations.

These findings highlight the potential of the NBA platform for multiplexed EV analysis. The combined approach provides a deeper understanding of the changes in EV subpopulations, either related to the appearance of a new EV population or to the modification of the existing one. The validation of particular EVs’ subpopulations through their biophysical and morphological properties offers a foundation for identifying biomarkers associated with cellular responses to cytotoxic stress.

## 5. Conclusions

This study explored the capabilities of the NBA platform to phenotype EVs derived from HMEC-1 cells, focusing on the impact of B[a]P-induced cytotoxicity on surface protein expression. A miniaturized biochip design was developed, enabling the simultaneous analysis of 11 ligands, including a negative control, on the same platform. SPRi experiments on the whole EV pool revealed significant differences in responses for Anti-CD36, Anti-CD81, and Anti-ApoA, suggesting their association with B[a]P exposure. Complementary AFM analysis and statistical evaluation highlighted size-related differences between treated and control EVs, identifying CD36, PS, and VEGFR1 as potential biomarkers for the cytotoxic condition. These findings suggest that B[a]P exposure not only alters the size of specific EV subpopulations but also selectively modifies the expression of several surface markers, indicating relative shifts in the phenotypic profile of 10k EVs. Moreover, the metrology investigation of specific EV subpopulations also made it possible to significantly highlight certain EV biomarkers linked to B[a]P exposure. The combined use of SPRi and AFM emphasizes the potential of lEV-specific markers to uncover phenotypic changes associated with cytotoxic stress, offering valuable insights into EV heterogeneity and their role in cellular stress responses.

This method is both comprehensive and highly versatile, making it easily adaptable to decipher other EV types and heterogeneous biological particles. With further expansion of the ligand panel, this platform has the potential to go beyond addressing biological questions, evolving into a powerful diagnostic tool for identifying disease-specific EV phenotypes and biomarkers.

## Figures and Tables

**Table 1:** List of chosen ligands with their grafting pH.

Antibodies/Protein Grafted	Manufacturer (Ref)	Grafting pH
Annexin-V	Biovision (1005-100), Nanterrre, France	7
Anti-CD63	Diaclone (857.770.000), Besançon, France	5.5
Anti-CD9	Abcam (Ab2215), Cambridge, UK	6
Anti-CD81	Invitrogen (MA1-10290), Illkirch-Graffenstaden, France	4
Anti-CD44	Biolegend (103001), San Diego, CA, USA	4.5
Anti-CD36	Invitrogen (MA5-30176)	4.5
Anti-NOX2	Abcam (EPR24537-56)	4.5
Anti-Enolase 1	Santa Cruz (SCBT SC-151013), Dallas, TX, USA	7
Anti-ApoA	Invitrogen (MIA1405)	4
Anti-VEGFR1	Abcam (ERP21886-207)	6
Anti-OVA (negative control)	Sigma-Aldrich (A6075), Lyon, France	4.5

**Table 2:** Summary of the Mann-Whitney U test results on large EVs.

**Ligands**	***p* Value**		**Significance**		Significance *
	Maximum Diameter	Height	Maximum Diameter	Height	Maximum Diameter + Height
Anti-CD36	0.031	0.001	+	+	**++**
Anti-VEGFR1	0.546	0.000	=	+	**+**
Annexin-V	0.08	0.002	=	+	**+**
Anti-CD44	0.795	0.089	=	=	**=**
Anti-CD63	0.173	0.111	=	=	**=**
Anti-NOX2	0.157	0.09	=	=	**=**
Anti-CD9	0.501	0.036	=	+	**+**
Anti-CD81	0.030	0.803	+	=	**+**
Anti-ApoA	0.277	0.843	=	=	**=**

* **++**: Significance based on maximum diameter and height; +: significance based either on maximum diameter or height; =: no difference.

## Data Availability

The original contributions presented in the study are included in the article; further inquiries can be directed to the corresponding author.

## Supplementary Materials

The following supporting information can be downloaded at: www.mdpi.com/article/10.3390/bios15020103/s1, Figure S1: Representative results obtained during the passivation step of a biochip with Rat Serum Albumin (RSA) leading to a validated biochip for the study. (a) Sensogram from one experiment during RSA (200 μg/mL) injection at 50 μL/min for 4 min; (b) the response obtained after RSA grafting and stabilization of the baseline. Figure S2: Representative results obtained during the passivation step of a biochip with Rat Serum Albumin (RSA) leading to a rejected biochip for the study. (a) Sensogram from one experiment during RSA (200 μg/mL) injection at 50 μL/min for 4 min; (b) the response obtained after RSA grafting and stabilization of the baseline. Figure S3: Split violin plot of EV size distribution: (a) maximum diameter (μm); (b) height (nm), where the middle dashed line is the median, and the outer dashed lines are the 25th and 75th percentiles. Control (blue) & Treated (orange) conditions.
